# Supplementary material for: “They are hard to navigate” exploring healthcare providers experiences managing Self-Injurious Behaviors among children with Autism Spectrum Disorder in Uganda
Source: PLOS Ment Health. 2026 Jun 4;3(6):e0000627. doi: 10.1371/journal.pmen.0000627 (PMC13235864; doi:10.1371/journal.pmen.0000627)
Supplement: S1 Code — (DOCX) [file pmen.0000627.s001.docx]

**Self-Injurious Behaviors Codebook**

| **Theme** | **Subtheme** | **Definition of subtheme** | **Quote from Transcripts** |
| --- | --- | --- | --- |
|  | healthcare providers perceptions | health care providers' perceptions are described as the healthcare providers' views, attitudes, and perspectives of SIBs | 1. Majority of them are egocentric; they want things there and then, yes, so you find that if you delay just for a minute, you just throw what I can call a tantrum and they'll just do it over and over until you'll be able to give them what they want or any other alternative to their situation.- **Occupational Therapist 2**  2. We have kids who are high-functioning autistic patients, and then there are those who are socially incompetent, like totally, and they are not verbal. They just throw tantrums, and they're aggressive.- **Pediatrician**  3. a child with autism, because of that hyposensitivity to pain, they end up into these self-injurious things so many times, injurious kind of behaviors whereby you just see a wound, you just see tissues have been damaged, body tissues have been damaged, and then you have to go all over again to see that you seek help.- **Psychologist**  4. And like I said, you have to think for them. It is more of your concern than their concern.- Psychologist  5. …early on in my profession, I thought that self-injurious behavior came with low IQ. – **Psychologist 3**  6. It could also be avoidance, avoid tasks, and seek attention.- Speech and Language therapist 2 |
| Perceptions and Manifestation of Self-Injurious Behaviors | Common forms | Healthcare providers described the common forms of SIBs as the different ways through which children with autism cause harm to themselves that they have seen or worked with. | 1. “The common self-injurious behaviors I've seen are head-banging. Another one was self-pinching… like the child would just start pinching themselves or peel their nails, and they keep peeling until you see that there is blood. Another one is eye-poking. A certain child I worked with used to really want to poke my eyes.” – **Occupational Therapist 1**  2. “Yes, I've seen plenty of students with autism banging their heads onto the walls, sometimes out of anger, sometimes out of happiness. They could be happy and they  would just decide to keep on banging. Majority of the ones that we see, they bite their skins to an extent that you might even see like their wrist or any joint is now hardened,  like the skin has hardened because they keep biting that area over and over again, of course due to several other reasons. One of the most common ones is hitting themselves  onto the ground, maybe out of anger or any other stuff, so they keep hitting themselves until you come for a rescue.” - **Occupational Therapist-2**  3. “I think the commonest I’ve seen is headbanging. It’s almost seen in every child with an autism spectrum disorder. I’ve also seen skin picking, scratching, pulling out hair, slapping oneself in the head, scratching their face and eyes.” – **Psychiatrist**  4. “So, the most common I've seen is hitting, self-hitting, usually on the head. So, I have seen them hit their heads, either slap themselves, or bang them onto hard surfaces, whether it's a wall, or it's someone else's head, or a table.” – **Speech and Language Therapist 1**  5. “Especially, hair-pulling, then banging their heads against the wall, eating non-food items. Then the other is really a child throwing themselves on the floor, and that is usually  looked at as a tantrum”- **psychologist 3**  6. I have seen children who head-bang. I've seen kids who bite themselves. I've seen kids  who cut their arms. -**Pediatrician** |
|  | Triggers and Antecedents | Healthcare providers described triggers and antecedents as the causes of SIBs among children with ASD | 1. “Most of these self-injurious behaviors, I've noticed, are among non-verbal children with autism. They're frustrated or you're engaging them and they're tired. They can't tell you, ‘I'm tired.’ That is their way of communicating—by hitting themselves on the wall or furniture.” – **Occupational Therapist 1**  2.“I think it’s mainly the first time they are trying to communicate something—their distress or something. Then they do the headbanging or something, and the parent has to figure out what the problem is.” – **Psychiatrist**  3. “She gets so frustrated when her parents don’t understand what she’s doing. She was cutting herself. At first, I thought it was attention-seeking behavior, but later I understood she was more interested in numbers and didn’t know how to express it.” – **Pediatrician**  4. It depends. There are most times when there are changes in routine or when the child wants to run away from a particular environment. Then there are also times when the  child is going to fall sick. They could have those temper outbursts, and that could trigger that to happen. - **Occupational Therapist 3**  5. Not that they want to head-bang, but it's repetitive behavior. It starts with banging on softer surfaces and then really banging, really, really hard. -**Pediatrician**  6. They commonly occur when the children are in distress, when they don't get their way or  they want to communicate something, but they're not able to communicate. -**psychiatrist**  7. I think the major cause is low or a lack of communication skills. Because ideally, where another child would say, I don't want to go, or I don't want to eat this, or leave me alone, I want to rest or something, this child does not have a means of communicating their needs. I think a lack of communication is the most common cause of Self-injurious Behaviors. -**Psychologist 2**  8. usually for the cases I have seen the behaviors are brought about by the tasks being difficult or them wanting to avoid them. -**Speech and Language Therapist 1**  9. sometimes I think it's probably because of boredom or trying to self-stimulate when they have nothing to do. -**Psychiatrist**  10. The repeated routine, which is so common with children with Autism, like when you're talking about a child on Autism Spectrum Disorder, we know that they have those things they come to really engage in and they repeatedly do them. So, when they start this self- injurious behavior, somehow, they fit into that repeated kind of routine, which is purposeless. -**Psychologist** |
| Interventions used in the management of SIBs | Medical Interventions | Healthcare providers described medical interventions as interventions that involved the use of medication to calm the child down/ Manage the symptoms of SIBs or ASD | 1. “In severe cases, we use low-dose antipsychotics like Risperidone, often for kids with ADHD or severe SIB. It’s not a cure—it’s just a bandage while we work on behavior.” – **Psychiatrist**  2. “Some children are given medication to calm them before therapy. It's usually used alongside occupational and behavioral therapies.” – Occupational Therapist  3. What I have seen in the children's clinics is some children with those challenging behaviors, they are given medication to first calm them down a bit. Most of the time, I see they are given a certain medicine, is it Risperidone. -**Occupational Therapist 1**  4. Usually the time when I have to go the route of meds is generally those who have comorbidities. Let's say they have self-injurious behavior, but maybe they also have ADHD. Usually we use antipsychotics, Risperidone, on a low dose, and see how that works. -**Psychiatrist**  5. I remember one was Adderall, but now they have lots of Adderall with other names around. But its main focus, there is also one called Concerta. Concerta is much given  with those with ADHD and Autism, while they are saying that it helps them to focus, which I don't believe that it helps them to focus because they will just hyper-focus like  this and even if you call them, they won't be turning -**Occupational Therapist 2**  6. We recommended Ritalin -**Psychologist 3** |
|  | Behavioral Interventions | Healthcare providers described behavioral interventions as any intervention that didn’t involve the use of medication | 1. If I know that someone has self-injurious behaviors, I will also try to protect them. Sometimes, it can be so too much that it might even require you to hold their hands. Just  hold them, reassure them if it requires me just to sing a song, just humming, just to make them calm down until maybe they get a little better. -Occupational Therapist 2  2. maybe if they want to communicate something give them alternatives listen to them use the picture communication use the sign language to make sure that you develop  understanding them and also develop an alternative communication to them as early as possible in order to replace the self-injurious behaviors. -**Occupational Therapist 2**  3. I like the distractive approach, you know? Play therapy is very helpful, doing art. I once saw this kid who was always throwing tantrums, like always throwing tantrums, and the  carrot-and-stick approach helped a lot. -**Pediatricia**  7. restraining is one of the things that we would do you sit behind the child and you hold  them and then you sing make them do what, distracting them -**psychologist 2**  8. One of the key things that I'm working with the children with autism is social skills training. So, we do lots of social stories, depending on the age. Social, we do experiential  social stories -psychologist 3  9. what I do most of the time is restraining. Yeah, restraining the hands, trying to calm the child down, and then trying to draw them back to the activity, because time out doesn't really work. -Speech Language Therapist 1  10. Restraining them. So, usually putting them in a corner. So, what we do is move away from the task, get them into a corner where they have limited movement, and restrain  their hands. And then have them calm down.- **Speech and language Therapist 1**  11. If they have long nails, I always make sure that every after three or four days, I trim them until they are not able to scratch themselves with those nails. If they have hair, they are so much into pulling their hair, I will give recommendations to the parent and the school to  always trim their hair to be low. Maybe until later when they learn self-regulatory mechanisms. -**Occupational Therapist 2**  13. managing the transitions. So, what happened is we came up with picture cards. And this, then, this. So, we would have a picture of swimming, then a picture of therapy. When we were leaving class, he's getting ready for swimming. You're like, we are going to go for swimming, and then we are going to do this. -**Psychologist 2**  14. |
|  | Protective aids and environmental modification |  | 1. When a child, was doing a head-banging, I started to think about a helmet. I thought about a helmet. As much as we could think about a helmet -**Psychologist 1**  2. sometimes if a child is injuring themselves, they are injuring their hands or they are injuring their body using their hands, we started splitting them, especially that hand. We could find that sometimes it is not both hands, but one hand is more into that, so you could end up trying to split that hand because when you split that hand and it is not  flexing, then it cannot make the fist. -**Psychologist 1**  3. he would bite all the nails, they were completely off. Now he could now start eating the skin of the palm and it could bleed dry it, to an extent that the palms became so hard and it was like the palms of a builder. So that was the first solution: gloves. Gloves every after eating food. Gloves after showering in the evening. That's what we would do. **Occupational Therapist 2**  4. for the one who used to pinch, I put masking tape on his arms so that when he pinches, because he used to really pinch hard, and you would see flesh, like a flesh wound. So, we cut off his nails, and we also put masking tape. -**Psychologist 2**  5. how I managed was, one, we got a safety helmet for that one child, the one who used to head bang the most. We got a safety helmet -**Psychologist 2**  6. 4. make sure they wear gloves. So, she uses socks. Just to prevent this child from scratching  themselves. -**Psychiatrist**  7. One of the things I have done is, the first thing is to remove, like if there are tables and  chairs, I remove the things that are movable. I make sure I put them away. **Occupational Therapist 1**  8. we cushion the room, cushion the floors, put those soft cushions. If the child wants to hit  themselves, there is a soft thing where they can do that, not injurious. **Occupational Therapist 1**  9. And the first thing that I do is to alter or to modify that environment. **Occupational Therapist 2**  10. But the first thing which I do, change the environment, move them away from a lot of  people because they could be stimulating them a lot or they could be making noise. I put  them in a safe space which is cool and quiet. Occupational Therapist 2  11. We try to identify what are the antecedents of the behavior, what are the consequences.  And then we try to see how we modify those things. How to work around to modify the  environment (Psychiatrist)  12. I stopped working with them from places that had chairs and tables, and would go to an  empty room that had nothing, but floor padding. So that even if they threw themselves  down, even if they banged their heads on the floor, I knew that it was safe. (Psychologist 2) |
| **Challenges working with children with SIBS** | Limited knowledge and skills | Healthcare providers described lack of skills as the lack exposure, experience and knowledge working with children with SIBs | 1. “We’re not really trained in detail… we met these behaviors in the field.” – Speech Therapist  2. “There are few specialists. Even if parents have money, they can’t find therapists. One left the country, and no one replaced them.” – **Psychiatrist**  3. I don't use ABA. I don't have training in ABA. I don't have specialized training in autism management. So, what I use is knowledge. I go from school and my experience working from one kid to another. - **Psychologist 3**  4. Well, lack of skills, that was the first challenge because after my training you've learnt about psychopathologies, you've learnt about issues but when you go in the field and you have a child who is banging their head and you're working with them and you're like, okay what do I do here?- **Psychologist 2**  5. it's always a struggle to get the specialists. So even if the parents had money, let's just assume, even getting specialists to give those services is quite tricky.- **Psychiatrists**  6. my success rate with such behaviors is usually low. It's usually low. And generally, for speech therapists, the speech therapists that I've worked with, behavior can be a major challenge for us.- **Speech and language therapist 1** |
|  | limited Resources | Healthcare providers described resources | 1. And some of those things are also expensive the materials used.  2. But sometimes the families are poor, they cannot afford all the things you're proposing and that can create a bit of frustration. - **Pediatrician**  3. there are tools, I mean the resources. I don't have them; the resources are not available -**Occupational Therapist 1**  4. there's a financial problem. So, they usually can't get, because sometimes some people can't get speech therapy. And they're really need speech therapy. – **Psychiatrist**  5. a lot of people come into the clinic and you tell them come after 2 weeks and they are like I don’t have transport money. So sometimes maybe you want to see the kid after two weeks but the parent says I cannot afford and you know there is nothing you can do so you push it to a month.- **Psychiatrist**  6. |
|  | Uncooperative parenting |  | 1. The first thing is parents, but now I can understand they are desperate and they want results day and then so they will scold you, they will blame you, they will do anything, they will be late, they will be inconsistent, but there's nothing to do.- **Occupational Therapist 2**  2. And again, still, the other hard part was coming in terms with the parents. Like at sometimes, you tell the parent, let’s try to avoid this and this, but if you feel like you want your child to have it, then maybe do it over the weekend so that during school time, we are having at least peaceful classes. But then their incidences, where you could find the child has come in with something that they're not supposed to be taking. **Occupational Therapist 2**  3. usually the ones that come back are those ones who have some medications or maybe they have epilepsy. But if you tell someone, okay, let's do this to come up with a plan for behavioral management, okay, let's do this and then come in after one month for us to see how it goes. And they're not going to do any medication, nothing. They don’t come back.- Psychiatrist  4. So, you would sit with them. Come up with a routine. But then they wouldn't follow it when you are not around. And sometimes you would have to train maids. Because the maids stay at home with the kids. So, you are training the caregivers. We would have sessions where we would train caregivers. But then the caregiver would say, the boss refused to do this. – Psychologist 2  5. The most frustrating bit is when you guide parents on what to do. They seem to be on board, but then back home, not much is done.- Psychologist 3  6. |
|  | **Healthcare providers frustration** |  | 1. of course, the frustration. We all want to work on someone and someone pulls out and we are okay. Now the frustration really comes in if someone is not pulling out. -Psychologist  2. The most frustrating bit is when you guide parents on what to do. They seem to be on board, but then back home, not much is done. - Psychologist 3  3. Not much, you know, working with these children, it's a long process. Like, you have to keep trying. Some days, the child will take about a week without maybe banging themselves. - Occupational Therapist  4. First of all, they are very hard to navigate. They are very hard to stop, and they're also very hard to work around with.- Speech and Language Therapist 1  5. Of course the frustration wasn't because the child is presenting that way the frustration was because the child is hurting themselves you know they are continuously banging this particular spot and they're getting bumps and still growing in there so as a person I would relate that they're feeling pain of course it has to be painful they are banging this particular bump and you're trying to stop them but they are not so that's the source of my frustration- Speech and Language Therapist 2  6. By experience, it is very exhausting. It is very tiring because you have to be watching them. Most of their work full time. You can only take a rest when they are actually sleeping.- Psychologist |
|  | **Misconceptions about SIBs** |  | 1. They think maybe the child has got a low IQ, maybe they are not really, they're not bright.- Occupational Therapist 3  2. I feel like that's the biggest misconception I come across. You have to explain to the parent that, you know what, these are not exactly bad manners of being in this clinic. It's a child trying to communicate. – Psychiatrist  3. People interpret them, or misinterpret them, maybe as something completely different, like the child was bewitched.- Psychologist  4. There's a school I went to, and the child was being called “mad.” And the child is mad, from the gate man to the head teacher. The child is mad. So, each time he got angry, he would go and punch. He could bang on a tree. And all they would do is to record him. – Psychologist 3  5. Initially, I thought that self-injurious behaviors were brought about by the discipline at home. So, thinking that, for example, maybe these kids at home are hit, so they pick it up and they start inflicting pain on themselves. – Speech and language Therapist 1  6. “Autism is highly stigmatized. One mother was battered because she had a child with autism. The father left, blaming her.” – Psychiatrist  7. We need to destigmatize autism—people still think it’s a curse or bad parenting.” – Pediatrician |
