# Supplementary material for: “They are hard to navigate” exploring healthcare providers experiences managing Self-Injurious Behaviors among children with Autism Spectrum Disorder in Uganda
Source: PLOS Ment Health. 2026 Jun 4;3(6):e0000627. doi: 10.1371/journal.pmen.0000627 (PMC13235864; doi:10.1371/journal.pmen.0000627)
Supplement: Attachment — Submitted filename: Response to Reviewers- Final(1).docx [file pmen.0000627.s003.docx]

**Journal Requirements**

|  | **Journal requirement/peer review comments** | **Response** | **Pg.** |
| --- | --- | --- | --- |
| 1 | - In the online submission form, you indicated that "Data is available upon request." All PLOS journals now require all data underlying the findings described in their manuscript to be freely available to other researchers, either  1. In a public repository,  2. Within the manuscript itself, or  3. Uploaded as supplementary information. | Data underlying the findings described in their manuscript will be uploaded as supplementary information |  |
| **2** | **Data Collection**   - The author should elaborate on how data saturation was achieved in this study. A clear description of the processes and indicators used to determine saturation would strengthen the methodological rigor. | The concept of data saturation was added to this section. See lines 128-130 |  |
| **3** | **Ethical Considerations**   - The manuscript indicates that recruitment was conducted through the dissemination of study flyers within healthcare professionals’ (HCPs) professional associations. However, it does not specify whether formal permission was obtained from these associations to facilitate recruitment and data collection. It would be beneficial to provide the names of the associations involved and clarify the approval processes followed. Furthermore, four interviews were conducted at participants’ workplaces. The manuscript does not explain how access to these workplaces was secured, nor whether institutional permission was granted. Clarification on the procedures followed to obtain organizational consent is necessary. | A section on study material dissemination has been included, detailing the relevant groups to which the study flyer was shared and the permissions sought. See lines 97-102  A section on how permission was obtained from participants. See lines 101-102. |  |
| **4** | **Table 2: Participant Demographics**   - In Table 2, the workplace for some participants is listed as “Clinical.” This designation is ambiguous and does not clearly convey the practice setting. It should be specified whether this refers to a private practice, hospital, clinic, or another healthcare environment. | The clinical designation has been changed to convey the more practical setting. See table 2 and lines 165-165. | **9** |
| **5** | - Two participants reportedly had specialized training in ASD and the management of SIBs. However, the findings do not indicate whether the management strategies implemented by these trained participants differed in effectiveness from those without such training. The manuscript should clarify the impact of specialized training on the management of SIBs and whether this influenced outcomes or approaches to care. | Thank you for pointing this out. However, this study did not aim to compare the effectiveness of management strategies between healthcare providers with specialized training and those without such training. Therefore, no analysis was conducted to determine differences in outcomes or approaches based on training. The study focused on exploring the overall experiences of healthcare providers managing self-injurious behaviors among children with ASD. |  |
| **6** | **Interpretation of the Findings**   - A deeper level of thematic analysis is required to strengthen the study’s theoretical grounding and contribution to the body of literature. For example, the statement: “Providers highlighted self-stimulating as being central to the development of SIBs. Notably, they identified boredom as contributing to persistent SIBs because most of these children have nothing to do,” reflects surface-level experiential reporting from healthcare professionals and lacks sufficient theoretical engagement. The findings would benefit from more robust conceptual interpretation and linkage to existing theoretical frameworks and empirical scholarship. | Thank you for highlighting the need for a deeper analysis. A deeper analysis of the findings was conducted and revealed two themes and four themes. These have been added to strengthen the findings. Additionally, theoretical examples have been provided throughout the results section ensuring that the findings are grounded in existing theory and literature. See results section |  |
| **7** | **Abstract**   - This qualitative study explored the experiences of healthcare providers managing self-injurious behaviors in children aged 5-12 years diagnosed with Autism Spectrum Disorder. Rather say This qualitative study explored the experiences of healthcare providers in managing self-injurious behaviors in children aged 5-12 years diagnosed with Autism Spectrum Disorder • Kindly indicate the study design and how many healthcare providers were interviewed. • What was the sampling strategy, like how were the healthcare providers selected? • Three core themes emerged from the data, including perceptions of self-injurious behaviors, management strategies, and providers' experiences managing self-injurious behaviors. The study was on experiences, hence the need to have themes that only emerge from experiences, not perceptions. Kindly relook at the core themes. • Keywords: Autism Spectrum Disorder; Self-Injurious Behaviors; healthcare Providers; Low-income Setting. The low income setting can not be a key word. Kindly add children, experiences and Uganda as key words. Also check how many keywords are required as per journal guidelines. | The sentence has been revised to read: *“This qualitative study explored the experiences of healthcare providers in managing self-injurious behaviors among children aged 5–12 years diagnosed with Autism Spectrum Disorder.”*  The abstract has been revised to specify that the study is a qualitative phenomenological study and that ten healthcare providers were interviewed.  The abstract has been revised to indicate that maximum variation purposive sampling was used to recruit healthcare providers.  The themes have been revised in the abstract to: (1) personal interpretation of SIBs and their triggers, (2) strategies used in managing SIBs, and (3) challenges encountered in managing SIBs.  The revised keywords are: Autism Spectrum Disorder; Self-Injurious Behaviors; Children; Healthcare Providers; Experiences; Uganda  See lines 22-352 | **2** |
| **8** | **Introduction**   - This section does not start with a definition. Rather start with the following, then definition can follow: SIBs, though not a symptom of Autism Spectrum Disorder (ASD), are highly prevalent among children with ASD (2). Currently, the prevalence of SIBs 43 among children with ASD is estimated at 42% (4). - Indicate if there are any studies done in other countries on the experiences of healthcare providers etc - Understanding their perspectives, management strategies, and experiences can inform the development of targeted training programs, supportive policies, and culturally relevant intervention strategies. The focus of the study is on experiences, not perspectives and management strategies. Kindly realign with what is in the topic. | The introduction section was modified as suggested. See line 42-46  We expanded the section to include studies examining HCPs’ experiences while caring for children with ASD. This includes literature from high income countries and Sub-Saharan Africa. We also clarify that the existing research rarely examines HCP’s experiences managing SIBs specifically which shows how important our study is in addressing this gap. See lines 60-66  The paragraph was rephrased to realign with the study topic. See lines 79-81. | **3-4** |
| **9** | **Methods and Materials**   - We sought to explore the perspectives and experiences of HCPs who support children diagnosed with ASD presenting with SIBs in Uganda. In this sentence, kindly remove the word perspectives because the focus is on experiences as stipulated in the topic. Again, the design used is specifically for lived experiences. Yes, these terms are used interchangeably but they are different. For instance, Experiences are the events a person lives through, while perspectives are the subjective lenses that are shaped by culture, background, and emotions. - Kindly give the detailed description of the setting in which the study was done - To be eligible for participation in the study, HCPs must have [1] had experiences supporting children diagnosed with ASD and presenting with SIBs, [2] possessed a minimum educational qualification equivalent to a diploma, [3] the ability to read and write in English, and [4] be capable of providing consent. HCPs who did not meet the above criteria were excluded. This should be put under the descriptions of the participants.   **Participants:**   - In this section, participants, sample and sampling were described, meaning the subheading should have the trio. Moreover, what determined for sample size for this study? - Participants: The central region is highly diverse and is home to Uganda’s capital city, Kampala. Kampala presented an appropriate setting for this study, as studies have shown that most specialized healthcare providers in Uganda are located within the central region (14,15); furthermore, the central region has been found to have the highest population of children diagnosed with ASD (16). This should be described under the setting, not under participants - We used maximum variation sampling to recruit participants from diverse settings within the central region of Uganda. This is incomplete description of the sampling. Maximum variation is a type of purposive sampling strategy and this need to come out. | The word “perspectives” has been removed to align the description with the study focus on experiences. See lines 82-91  The participant eligibility criteria have been moved to the Participants section. See line 107-110  The participant eligibility criteria have been moved to the Participants section  The subheading has been revised to “Participants, Sample, and Sampling.” | **5** |
|  | **Data Collection**   - This section is starting with data collection procedure which is not correct. First indicate the data collection tool used including the questions asked. Then, describe the data collection procedure followed - On data analysis, thematic approach was used. This is a phenomenological study, why was this approach used instead of Interpretative phenomenological analysis (IPA)? - The Trustworthiness of qualitative data in terms of dependability, transferability and confirmability was not explained for this study. Only credibility was described. Kindly add. | The section has been modified to begin with the data collection tool followed by the questions asked and finally the data collection procedure. See line 113-117  In this study, thematic analysis was preferred over Interpretative phenomenological analysis because it offers a structure yet flexible framework for identifying, analyzing, and interpreting patterns of meanings within datasets (Ahmed et al., 2025). Additionally, IPA focuses on individual lived experiences and idiographic depth, whereas thematic analysis focuses on individual meanings, context, and interpretation, which was the focus of this study. | **6** |
|  | **Results**   - Table 4: Summary of Themes and Codes: In the table, there are no codes, but themes and subthemes. Again, did you want to say subthemes and codes. Because it is the subthemes that you describe in the content. The main theme becomes experiences. - Three main themes emerged from the data, including: [1] Perceptions of SIBs, [2] Techniques and strategies used to manage SIBs, [3] Experiences managing SIBs. Relook into these emerging themes. First, they should all emanate from the main theme of experiences. Secondly, there is need to rename that subtheme of perceptions because as I said before, experiences are not perceptions, rather say personal interpretation of SIBs and their triggers as the subtheme. On number 3, is it experiences or challenges in managing SIBs as an experience? Again, the nature of the challenges cannot be considered under one theme, they need to be standalone. For instance, societal stigma and misconceptions should be a standalone theme which should be explained differently. From the sample table below, each of the subtheme below need to be described in detail and two quotations for each theme should be given. | Thank you for this observation. Yes, we agree that the main theme is experiences and what is presented in Table 4 are sub-themes and codes. This has been revised. See line 177  This section has been clarified. Sub-theme 1 renamed to ‘Personal interpretation of SIBS and their triggers’ to reflect experiences rather than perceptions and stigma separated from misconceptions with quotes added under each code. See lines 177-178 and 350-366 | **11 and 19** |
|  | **Limitations**   - Kindly include information bias and researcher bias as possible limitations of this study. - Although these factors may limit the generalizability of our findings, the study nonetheless provides valuable preliminary insights into the experiences of HCPs working with children on the autism spectrum who present with self-injurious behaviors (SIBs), offering a foundation for future research in this population. This is a qualitative study, you don’t talk of generalisability, but transferability. - Finally, as children with ASD and their parents were not included, the study cannot provide a complete representation of all perspectives regarding SIBs, interventions, and related challenges. The study was on experiences, not perspectives. | Information on researcher and information bias as potential limitations have been included in the limitations section see lines 465-472.  This has been changed to remove generalizability and include transferability. See lines 469-471.  Thank you for this suggestion, the sentence has been modified, and the term "perspectives" has been removed and replaced with "experiences," the focus of this study. See lines 476-478. | **24** |
