# Supplementary material for: “They are hard to navigate” exploring healthcare providers experiences managing Self-Injurious Behaviors among children with Autism Spectrum Disorder in Uganda
Source: PLOS Ment Health. 2026 Jun 4;3(6):e0000627. doi: 10.1371/journal.pmen.0000627 (PMC13235864; doi:10.1371/journal.pmen.0000627)
Supplement: Attachment — Submitted filename: Response to Reviewers(1).docx [file pmen.0000627.s004.docx]

Reviewers Responses

| No | Peer Reviewer suggestion | Response | Pg. |
| --- | --- | --- | --- |
| 1. | The Trustworthiness of  qualitative data in terms of  dependability, transferability  and confirmability was not  explained for this study. Only  credibility was described. | Thank you for this suggestion we have included the processes through which we ensured the trustworthiness of our research. | 9-10 |
| 2 | Please review your reference list to ensure that it is complete and correct. If you have cited papers that have been retracted, please include the rationale for doing so in the manuscript text, or remove these references and replace them with relevant current references. Any changes to the reference list should be mentioned in the rebuttal letter that accompanies your revised manuscript. If you need to cite a retracted article, indicate the article’s retracted status in the References list and also include a citation and full reference for the retraction notice. | The reference section was reviewed and checked to ensure adherence to the journal requirement | 30-37 |
